# Supplementary material for: Genetic Events Inhibiting Apoptosis in Diffuse Large B Cell Lymphoma
Source: Cancers (Basel). 2021 Apr 30;13(9):2167. doi: 10.3390/cancers13092167 (PMC8125500; doi:10.3390/cancers13092167)
Supplement: Supplementary file 1 [file cancers-13-02167-s001.zip › cancers-1200904-supplementary.pdf]

Table S1. Ongoing clinical trials investigating therapies modulating apoptosis in DLBCL

DLBCL type	Investigational therapy	Trial phase	ClinicalTrials.gov Identifier
BCL2-positive DLBCL	Venetoclax + polatuzumab vedotin + R-CHP	Ib	NCT04790903
Double hit / double expressor lymphoma	Venetoclax + R-CHOP or DA-EPOCH-R	II/III	NCT03984448
DLBCL after ASCT	Tislelizumab maintenance	III	NCT04789434
EBV-positive DLBCL	Sintilimab + R-CHOP	II	NCT04181489
High-risk DLBCL	Nivolumab + DA-EPOCH-R	II	NCT03749018
Double/triple hit lymphoma	DA-EPOCH-R followed by nivolumab consolidation	II	NCT03620578
DLBCL	Sintilimab + R-CHOP	II	NCT04023916
ABC-DLBCL	Ibrutinib + R-CHOP followed by ibrutinib maintenance	II	NCT03731234
High-risk DLBCL	Ibrutinib + bortezomib + R-CHOP	I/II	NCT03129828
High-risk DLBCL	Ibrutinib + R-CHOEP	II	NCT03399513
DLBCL	Ibrutinib + lenalidomide + chidamide + decitabine + R-CHOP	II	NCT04025593
Non-GCB DLBCL	Ibrutinib + CA-4948 (IRAK4 inhibitor, toll-like receptor pathway suppressor)	I	NCT03328078
Double expressor DLBCL	Lenalidomide + R-CHOP	II	NCT04842487
DLBCL (age > 75 and unfit for conventional therapy)	Lenalidomide + zanubrutinib + rituximab	II	NCT04460248
High-risk DLBCL	Lenalidomide + tafasitamab + R-CHOP	III	NCT04824092
MYC-rearranged DLBCL	Lenalidomide + DA-EPOCH-R	I/II	NCT04432714
DLBCL (age > 80)	Lenalidomide + rituximab	II	NCT04622579
DLBCL	Lenalidomide + R-CHOP	II	NCT04214626
Double expressor DLBCL	Lenalidomide + R-CHOP	II	NCT04164368
Double hit lymphoma	Lenalidomide + DA-EPOCH-R	I/II	NCT02213913
DLBCL	DLCL002 protocol	II	NCT03837873
rrDLBCL + previously untreated DLBCL	Tafasitamab + lenalidomide +/- R-CHOP (if previously untreated)	I	NCT04661007
rrDLBCL	Venetoclax + R-ICE	I/II	NCT03064867
rrDLBCL	Venetoclax + selinexor	Ib	NCT03955783

rrDLBCL	Selinexor + various combinations including venetoclax, lenalidomide, and ibrutinib	I/II	NCT04607772
rrDLBCL	ViPOR-P	I	NCT04739813
rrDLBCL	ViPOR	Ib/II	NCT03223610
rrDLBCL	Venetoclax + lenalidomide + obinutuzumab	I	NCT02992522
rrDLBCL	Venetoclax + LOXO-305 (BTK inhibitor) +/- rituximab	I/II	NCT03740529
rrDLBCL	Venetoclax + ABBV-621 (TRAIL receptor agonist)	I	NCT03082209
rrDLBCL	Anti-CD19 / CD20 CAR T cells	I	NCT04215016
rrDLBCL	Anti-CD19 / CD20 CAR T cells	II	NCT04792489
rrDLBCL	Anti-CD19 CAR T cells	I	NCT03960840
rrDLBCL	Anti-CD19 CAR T cells + tislelizumab	I	NCT04381741
rrDLBCL	Anti-CD19 / CD20 CAR T cells	II	NCT04844866
rrDLBCL	Anti-CD20 CAR T cells	I	NCT04316624
rrDLBCL	Anti-CD19 / CD22 CAR T cells	I/II	NCT03287817
rrDLBCL	Apamistamab followed by anti-CD20 CAR T cells	I	NCT04512716
rrDLBCL	Anti-CD19 / CD20 CAR T cells	I	NCT04486872
Refractory DLBCL	Anti-CD19 CAR T cells	I	NCT04812691
rrDLBCL	Anti-CD22 CAR T cells	I	NCT04088890
rrDLBCL	Anti-CD30 CAR T cells	I	NCT04526834
rrDLBCL	Anti-CD19 CAR T cells	I	NCT03720457
rrDLBCL	Anti-CD20 CAR T cells	I	NCT04176913
rrDLBCL	Anti-CD19 CAR T cells	I	NCT04026100
rrDLBCL	Anti-CD19 CAR T cells	II	NCT04089215
rrDLBCL	Anti-CD19 CAR T cells + acalabrutinib	I/II	NCT04257578
rrDLBCL	Anti-CD19 CAR T cells + cyclophosphamide + fludarabine	I	NCT04049513
rrDLBCL	Anti-CD19 CAR T cells	I	NCT04464200
rrDLBCL	Anti-CD19 / CD22 CAR T cells	I	NCT03233854
rrDLBCL	Anti-CD19 / CD20 CAR T cells + cyclophosphamide + fludarabine	I	NCT04007029
rrDLBCL	Anti-CD19 CAR T cells	I	NCT04844086

rrDLBCL	Anti-CD19 CAR T cells	I	NCT03103971
rrDLBCL	Anti-CD19 CAR T cells + cyclophosphamide + fludarabine	I/II	NCT03676504
rrDLBCL	Anti-CD20 CAR T cells + cyclophosphamide +/- fludarabine	I/II	NCT03277729
rrDLBCL	Anti-CD5 CAR T cells + cyclophosphamide +/- fludarabine	I	NCT04767308
rrDLBCL	Anti-CD19 CAR T cells + cyclophosphamide + fludarabine	I	NCT04545762
rrDLBCL	Anti-CD19 CAR T cells	I	NCT04231747
rrDLBCL	Anti-CD19 CAR T cells + cyclophosphamide + fludarabine	I/II	NCT04836507
rrDLBCL after HSCT	Anti-CD19 CAR T cells + cyclophosphamide + fludarabine	I	NCT03579888
rrDLBCL	Anti-CD19 CAR T cells + SWI019 (anti-CD19 antibody)	I	NCT04450069
rrDLBCL	Anti-CD19 CAR T cells	I	NCT02631044
rrDLBCL	Anti-CD19 CAR T cells + durvalumab	Ib	NCT02706405
rrDLBCL	Anti-CD19 CAR T cells + pembrolizumab	I	NCT03630159
rrDLBCL	Anti-CD20 CAR T cells+ cyclophosphamide + fludarabine + bendamustine	I	NCT04735471
rrDLBCL	Anti-CD19 CAR T cells +/- platinum-based immunochemotherapy + lymphodepleting chemotherapy	III	NCT03570892
rrDLBCL after CAR T cell therapy	Glofitamab + obinutuzumab	II	NCT04703686
rrDLBCL	Blinatumomab + lenalidomide	I	NCT02568553
rrDLBCL after ASCT	Tislelizumab maintenance	III	NCT04799314
rrDLBCL	Sintilimab + R-GemOx	II	NCT04659434
rrDLBCL	Nivolumab + tumor associated antigen specific T cells (TAA-T)	I	NCT03843294
rrDLBCL	Toripalimab + rituximab	II	NCT04425824
rrDLBCL	Pembrolizumab + CXD101 (histone deacetylase inhibitor)	I/II	NCT03873025
EBV-positive rrDLBCL	Tislelizumab + zanubrutinib	II	NCT04705129
Richter's transformation (previously treated)	Nivolumab + copanlisib	I	NCT03884998

rrDLBCL	Pembrolizumab + dendritic cell therapy + cryosurgery	I/II	NCT03035331
PD-L1 gene-altered rrDLBCL	Pembrolizumab	II	NCT03990961
rrDLBCL	STI-3031 (anti-PD-L1 antibody)	II	NCT03999658
rrDLBCL	Durvalumab + radiotherapy	I	NCT03610061
rrDLBCL	Ibrutinib + R-ICE	II	NCT02955628
Non-GCB rrDLBCL	Ibrutinib + abexinostat	I	NCT03939182
rrABC-DLBCL	Ibrutinib + ASCT	III	NCT02443077
rrDLBCL	Ibrutinib + loncastuximab tesirine	I/II	NCT03684694
rrDLBCL	Ibrutinib + bendamustine + rituximab	II	NCT02747732
rrDLBCL	Ibrutinib + selinexor	I	NCT02303392
rrDLBCL	Ibrutinib + pevonedistat	I	NCT03479268
rrDLBCL	Lenalidomide + rituximab + brentuximab vedotin	III	NCT04404283
rrDLBCL	Lenalidomide + tislezumab	Ib/II	NCT04796857
rrDLBCL (age > 75)	Lenalidomide + rituximab	II	NCT04113226
rrDLBCL	Lenalidomide + zanubrutinib +/- rituximab	I	NCT04436107
rrDLBCL	Lenalidomide + R-ICE	I/II	NCT02628405
rrDLBCL	Lenalidomide + pembrolizumab + rituximab	II	NCT02446457
rrDLBCL	Lenalidomide + nivolumab	I/II	NCT03015896
rrDLBCL	NX-2127 (BTK degrader)	I	NCT04830137

Data obtained from Clinicaltrials.gov
R-CHP: rituximab, cyclophosphamide, doxorubicin, prednisone
R-CHOP: rituximab, cyclophosphamide, doxorubicin, vincristine, prednisone
DA-EPOCH-R: dose-adjusted etoposide, prednisone, vincristine, cyclophosphamide, doxorubicin, rituximab
ASCT: autologous stem cell transplant
R-CHOEP: rituximab, cyclophosphamide, doxorubicin, vincristine, etoposide, prednisone
R-ICE: rituximab, ifosfamide, carboplatin, etoposide
HSCT: hematopoietic stem cell transplant
R-GemOx: rituximab, gemcitabine, oxaliplatin
ViPOR-P: venetoclax, ibrutinib, prednisone, obinutuzumab, lenalidomide, polatuzumab vedotin
